# The Effect of Percutaneous Coronary Intervention on Patients with Acute Myocardial Infarction and Cardiogenic Shock Supported by Extracorporeal Membrane Oxygenation

**DOI:** 10.31083/j.rcm2512449

**Published:** 2024-12-23

**Authors:** Yan Wang, Hongfu Fu, Jin Li, Haixiu Xie, Chenglong Li, Zhongtao Du, Xing Hao, Hong Wang, Liangshan Wang, Xiaotong Hou

**Affiliations:** ^1^Center for Cardiac Intensive Care, Beijing Anzhen Hospital, Capital Medical University, 100029 Beijing, China

**Keywords:** cardiogenic shock, extracorporeal membrane oxygenation, percutaneous coronary intervention

## Abstract

**Background:**

Patients suffering from acute myocardial infarction complicated by cardiogenic shock (AMICS), who undergo veno-arterial extracorporeal membrane oxygenation (VA-ECMO) therapy, typically exhibit high mortality rates. The benefits of percutaneous coronary intervention (PCI) in these patients remains unclear. This study aims to investigate whether PCI can mitigate mortality among patients with AMICS supported by ECMO.

**Methods:**

Data from patients ≥18 years, who underwent VA-ECMO assistance in China between January 1, 2017, and June 30, 2022, were retrieved by searching the Chinese Society of Extracorporeal Life Support (CSECLS) Registry. A total of 1623 patients were included and categorised based on whether they underwent PCI. Using propensity score matching, 320 patient pairs were successfully matched. The primary outcome was in-hospital mortality rate. The secondary outcomes included VA-ECMO duration, Hospital stay, ECMO weaning and ECMO related complications.

**Results:**

In the cohort of 1623 patients, 641 (39.5%) underwent PCI. Upon conducting multivariate logistic regression analysis, it was observed that those who underwent PCI had a lower prevalence of hyperlipidemia (13.1% versus [vs.] 17.8%), chronic respiratory disease (2.5% vs. 4.3%) and lower lactic acid (5.90 vs. 8.40). They also had a more significant history of PCI (24.8% vs. 19.8%) and were more likely to be smokers (42.6% vs. 37.0%). Patients in the PCI group exhibited lower in-hospital mortality before and after matching (40.3% vs. 51.6%; *p* = 0.005), which persisted in multivariable modeling (adjusted odds ratio [aOR]: 0.69; 95% confidence interval 0.50–0.95; *p* = 0.024). Patients who received PCI were more successfully weaned from ECMO (88.6% vs. 75.8% before matching). PCI was not a risk factor for ECMO related complications.

**Conclusions:**

Among patients who received ECMO support for AMICS, PCI was associated with a lower rate of in-hospital mortality.

## 1. Introduction 

Approximately 3–10% of patients who experience acute myocardial infarction (AMI) also experience cardiac arrest or cardiogenic shock (CS), which is associated with high in-hospital mortality rates [1, 2, 3]. Acute myocardial infarction complicated by cardiogenic shock (AMICS) is a complex clinical syndrome characterized by cardiac contractile dysfunction, resulting in the left ventricle being unable to maintain adequate cardiac output, therefore leading to insufficient perfusion of the surrounding tissues (decreased urine output, mental state change, or cold limbs) [4]. Timely emergency revascularization is a crucial intervention for AMICS, aimed at preventing permanent ischemic damage and salvaging the affected myocardium [5]. Worldwide, the application of percutaneous coronary intervention (PCI) is currently on the rise owing to the anatomical and clinical complexity of coronary artery disease (CAD) [6, 7]. According to myocardial reconstruction guidelines [8], coronary artery bypass grafting (CABG) is the first-line revascularization strategy recommended for patients with multivessel CAD. However, due to severe clinical manifestations and complex anatomical presentations, some patients may not be suitable candidates for CABG. Currently, CABG is seldom performed in patients with cardiogenic shock, as PCI stands as the predominant revascularization approach in such cases [9].

The ‘Should We Emergently Revascularize Occluded Coronaries for Cardiogenic Shock’ (SHOCK) trial, conducted approximately 20 years ago, was a randomized controlled trial (RCT) that demonstrated that early revascularization in patients with AMICS was beneficial for 6-month survival [5]. Since then, early revascularization of culprit blood vessels has become the cornerstone of treatment for patients with AMICS. Patients with AMICS are at high risk for decompensation due to preexisting left ventricular dysfunction, high comorbidities, multiple vessel lesions, and complex coronary artery anatomy. However, a study investigating the characteristics of patients with AMICS found that patients included in RCTs underwent more active treatment, such as PCI, than patients in registered institutions [10]. Although extracorporeal membrane oxygenation (ECMO) can provide hemodynamic support for patients with AMICS, a survey addressing the application of ECMO in patients with AMI in the United States found that only 39.3% underwent PCI [11]. Patients undergoing ECMO for frequently present with myocardial systolic dysfunction [4], and while PCI is not a universal intervention for all such patients. Is there a benefit of PCI in patients with AMICS supported by ECMO? This study hypothesizes that PCI for AMICS patients supported by ECMO would lead to improved clinical outcomes.

## 2. Methods

### 2.1 Data Source

Research data were obtained from the Chinese Society of Extracorporeal Life Support (CSECLS) Registry. Patient characteristics, pre-ECMO indicators, adverse events, veno-arterial extracorporeal membrane oxygenation (VA-ECMO) duration, Hospital stay, weaning of ECMO, in-hospital mortality rate and ECMO related complications were extracted using a standardized data collection form. Clinical diagnoses and comorbidities are reported with International Classification of Diseases, 9th and 10th Revisions—Clinical Modification (ICD-9/10-CM) codes. This study was approved by the Research Ethics Board of the Beijing Anzhen Hospital (2024165x).

### 2.2 Study Population

Adults (≥18 years of age) undergoing VA-ECMO between January 1, 2017, and June 30, 2022 were included. Patients who underwent multiple runs of ECMO were excluded. Though the use of multiple runs of ECMO is growing, outcomes remain poor for most cohorts. Survival decreases with each additional run [12]. We also excluded patients with central cannulation. Prior research has demonstrated that peripheral cannulation is independently linked to lower in-hospital mortality when compared to central cannulation in patients with CS [13]. Moreover, patients with non-ischemic heart disease and those undergoing CABG were also excluded, as their disease profiles differ from the primary study cohort.

### 2.3 Baseline Characteristics and Outcomes

Patient baseline characteristics included age, sex, body mass index (BMI), medical history, and general situation before ECMO. Chronic respiratory diseases include chronic obstructive pulmonary disease (COPD), asthma, and bronchiectasis. Hypertension, diabetes, hyperlipidemia and heart failure are reported with International Classification of Diseases, 9th and 10th Revisions—Clinical Modification (ICD-9/10-CM) codes. Chronic kidney disease (CKD) is characterized by a persistent decline in kidney function, typically indicated by a glomerular filtration rate (GFR) below 60 mL/min per 1.73 m^2^, or the presence of markers of kidney damage, or both. This condition persists for a minimum duration of 3 months, irrespective of the underlying causative factors. Cerebrovascular accidents include cerebral hemorrhage and cerebral infarction. Mean arterial pressure (MAP), potential of hydrogen (PH) and lactates are the worst data within 6 hours before starting ECMO assistance. The primary outcome was in-hospital mortality rate. Secondary outcomes included VA-ECMO duration, Hospital stay, weaning of ECMO and ECMO-related complications, such as haemorrhage, limb ischaemia, pulmonary infection, neurological complications, and continuous renal replacement therapy (CRRT). Haemorrhagic complications were defined on the basis of the registry definitions of the Extracorporeal Life Support Organization (ELSO) as any haemorrhage requiring surgical intervention or a red-blood-cell transfusion >20 mL/kg/24 h of packed red blood cells or >3 units of packed red blood cells/24 h per calendar day [14]. Significant neurological complications like seizures, ischaemic strokes and intracranial haemorrhage were counted.

### 2.4 Statistical Analysis

Continuous variables such as age, BMI, MAP, lactates, and PH have been found to follow non-normal distributions through normality tests. Categorical variables are expressed as count (percentage [%]) and continuous variables as median (interquartile range [IQR]). Differences between groups were tested using the Wilcoxon rank-sum test for continuous variables and the fisher’s exact or chi-squared test for categorical variables. A regression analysis was conducted for multivariable modeling, purposefully selecting statistically significant (with *p*-values less than 0.10) and clinically relevant variables that could potentially predict in-hospital mortality. Propensity score matching was conducted between the PCI and no-PCI groups using the estimated propensity scores. All baseline characteristics were incorporated as matching variables to control for confounding factors. A one-to-one propensity score-matched analysis was carried out through nearest-neighbour matching, with a caliper set at 0.02 standardized difference (SD) of the combined propensity scores. And all variables in the baseline data were accounted for in the matching process. Following the matching, covariates with standardized mean differences of 0.10 or greater after matching were adjusted for in subsequent logistic regression models. Kaplan–Meier survival curves were drawn to illustrate survival trends. Differences with *p* < 0.05 were considered to be statistically significant. All statistical analyses were performed using SPSS version 26.0 (IBM Corporation, Armonk, NY, USA).

## 3. Results 

### 3.1 Patient Characteristics

**Fig. 1.**
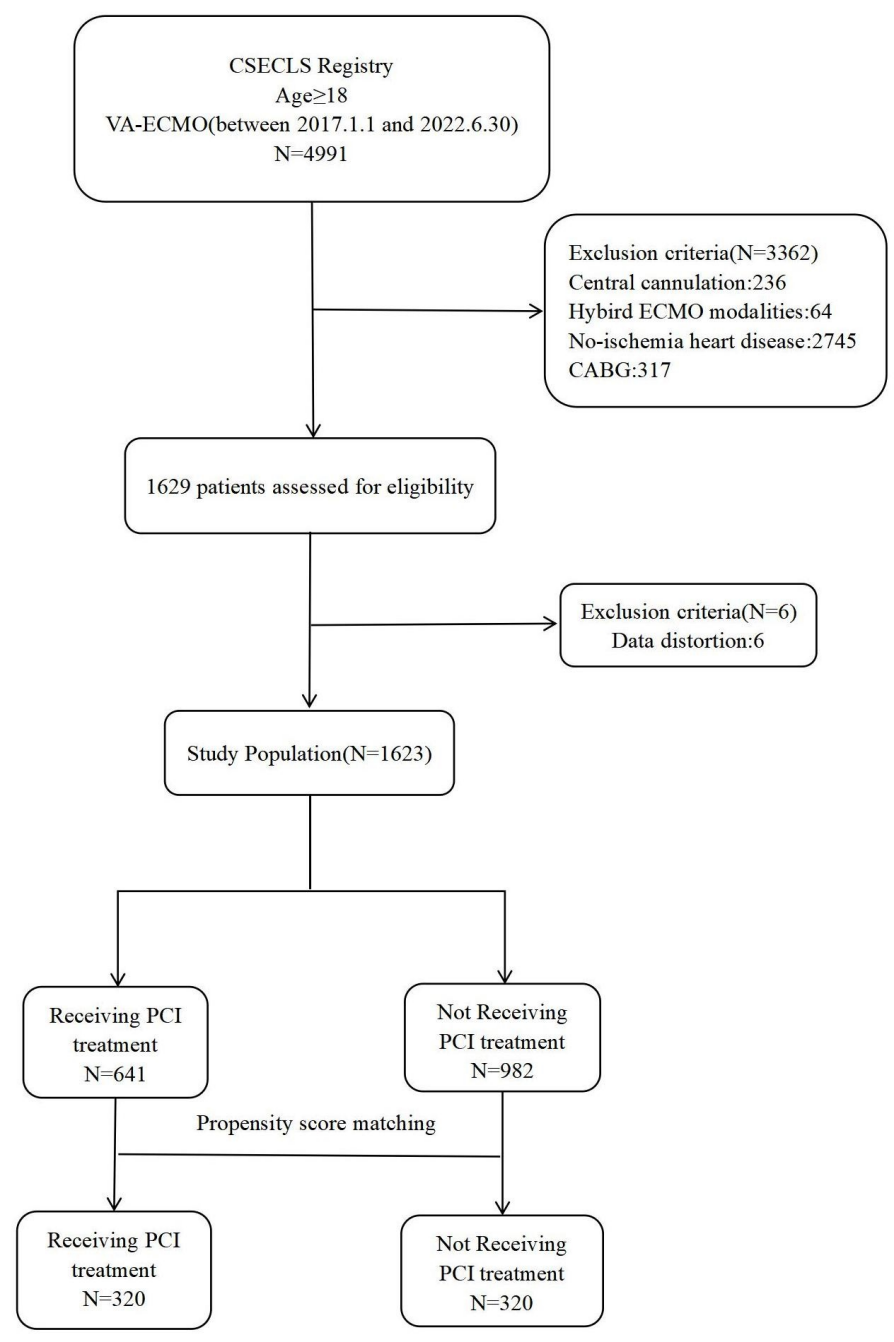
**A flow chart of the selection process for patients**. CSECLS, Chinese Society of Extracorporeal Life Support; VA-ECMO, venoarterial extracorporeal membrane oxygenation; CABG, coronary artery bypass grafting; PCI, percutaneous coronary intervention.

During the study period, 4991 patients who underwent VA-ECMO were identified. Of them, 1623 were eligible for the current investigation, including 641 who underwent PCI and 982 who did not (Fig. 1). One-to-one matching with propensity score matching was employed to generate 320 pairs.

The baseline characteristics of the unmatched and propensity score-matched groups are presented in Table 1. After propensity score matching, some indicators (age, sex, previous PCI and intra-aortic balloon pump (IABP)) remained imbalanced and were included in the logistic multivariate regression analysis of outcomes. In the PCI group, patients had a more frequent history of PCI procedures and were more likely to be smokers compared to the non-PCI group. Patients in the no-PCI group had a higher prevalence of comorbidities, including hyperlipidemia and heart failure, compared to the PCI group. Furthermore, 52.6% of patients in the no-PCI group suffered from cardiac arrest, a significantly higher percentage compared to the PCI group, where the incidence was 39.6% (*p* < 0.001). There were also significant differences in MAP, PH, and lactate between the two groups of patients. A logistic multivariate regression analysis was performed to assess the association between receiving PCI treatment and various factors, as presented in Table 2. Patients with hyperlipidemia, chronic respiratory disease, high lactate levels were less likely to undergo PCI. Patients with AMICS and ECMO support who have a history of PCI treatment and smoking are more likely to undergo PCI treatment. The population characteristics were consistent with those of previous studies [7, 15].

**Table 1.** **Baseline characteristics of the patients in the unmatched and propensity-matched groups**.

Variables [median (IQR)/n (%)]	Unmatched groups				Matched groups			
	PCI	No-PCI	*p*	SMD	PCI	No-PCI	*p*	SMD
	(N = 641, 39.5)	(N = 982, 60.5)			(N = 320)	(N = 320)		
Age (years)	61.0 (52.5, 69.0)	61.0 (51.0, 68.0)	0.198	0.069	61.0 (52.0, 69.0)	58.0 (48.0, 66.0)	0.004	0.234
Sex (male), n (%)	525 (81.9)	796 (81.2)	0.696	0.008	267 (83.4)	279 (87.2)	0.219	–0.107
BMI (kg/m^2^)	24.2 (22.2, 25.8)	24.0 (22.5, 25.7)	0.476	0.033	24.2 (22.2, 25.7)	24.2 (22.2, 25.9)	0.781	–0.029
Previous PCI, n (%)	154 (24.8)	190 (19.8)	0.021	0.118	82 (25.7)	67 (21.0)	0.190	0.112
Previous MI, n (%)	135 (21.1)	225 (22.9)	0.391	–0.049	71 (22.2)	72 (22.5)	1.000	–0.008
Smokers, n (%)	273 (42.6)	363 (37.0)	0.024	0.076	147 (45.9)	155 (48.4)	0.579	–0.050
Hypertension, n (%)	319 (49.8)	508 (51.7)	0.439	–0.040	160 (50.0)	148 (46.3)	0.384	0.075
Diabetes, n (%)	204 (31.8)	301 (30.7)	0.620	0.022	99 (30.9)	95 (29.7)	0.796	0.027
Hyperlipidemia, n (%)	84 (13.1)	175 (17.8)	0.010	–0.139	42 (13.1)	41 (12.8)	1.000	0.009
Heart failure, n (%)	78 (12.2)	156 (15.9)	0.042	–0.114	46 (14.4)	37 (11.6)	0.347	0.085
Cerebrovascular accident, n (%)	43 (6.7)	71 (7.2)	0.765	–0.018	15 (4.7)	14 (4.4)	1.000	0.016
Chronic respiratory disease, n (%)	16 (2.5)	42 (4.3)	0.074	–0.059	7 (2.2)	6 (1.9)	1.000	0.075
Chronic kidney disease, n (%)	25 (3.9)	26 (2.7)	0.190	0.070	9 (2.8)	7 (2.2)	0.801	0.040
IABP, n (%)	256 (39.9)	369 (37.6)	0.348	0.041	130 (40.6)	197 (61.6)	<0.001	–0.427
Cardiac arrest, n (%)	254 (39.6)	516 (52.6)	<0.001	–0.260	138 (43.1)	140 (43.8)	0.579	–0.012
PH	7.28 (7.11, 7.41)	7.21 (7.06, 7.35)	<0.001	0.068	7.3 (7.1, 7.4)	7.3 (7.1, 7.4)	0.488	0.022
MAP (mmHg)	52.7 (23.3, 65.7)	46.2 (0.0, 60.0)	<0.001	0.264	51.3 (0.0, 64.0)	50.0 (20.6, 62.8)	0.445	0.044
Lactates (mmol/L)	5.9 (2.3, 11.1)	8.4 (4.0, 13.3)	<0.001	–0.306	6.5 (2.5, 11.9)	7.0 (3.2, 11.2)	0.410	–0.027

Data are presented as medians (25th–75th percentile) or n (%). IQR, interquartile range; BMI, body mass index; PCI, percutaneous coronary intervention; MI, myocardial infarction; IABP, intra-aortic balloon pump; PH, potential of hydrogen; MAP, mean arterial pressure; SMD, standardized mean differences.

**Table 2.** **Multivariate logistic regression analysis of baseline characteristics of patients undergoing PCI treatment before matching**.

	Univariate analysis		Multivariate analysis	
	Odds ratio	*p*	Odds ratio	*p*
Age	1.00 (0.99, 1.01)	0.172	1.10 (0.99, 1.02)	0.184
Gender (male)	0.95 (0.73, 1.22)	0.669	1.06 (0.70, 1.59)	0.779
BMI (kg/m^2^)	1.01 (0.98, 1.04)	0.514	0.97 (0.72, 1.29)	0.952
Previous PCI	1.32 (1.04, 1.69)	0.021	1.65 (1.17, 2.33)	0.004
Previous MI	0.90 (0.70, 1.14)	0.380		
Smokers	1.27 (1.04, 1.56)	0.020	1.49 (1.10, 2.02)	0.011
Hypertension	0.92 (0.75, 1.13)	0.414		
Diabetes	1.05 (0.85, 1.31)	0.639		
Hyperlipidemia	0.69 (0.52, 0.99)	0.040	0.57 (0.38, 0.86)	0.008
Heart failure	0.74 (0.55, 0.99)	0.040	0.71 (0.47, 1.08)	0.110
Cerebrovascular accident	0.92 (0.62, 1.37)	0.690		
Chronic respiratory disease	0.57 (0.32, 1.03)	0.063	0.36 (0.16, 0.86)	0.021
Chronic kidney disease	1.49 (0.85, 2.60)	0.162		
IABP	1.11 (0.90, 1.36)	0.339		
Cardiac arrest	0.59 (0.49, 0.73)	<0.001	1.03 (0.71, 1.51)	0.886
PH	1.24 (0.85, 1.81)	0.269		
MAP (mmHg)	1.01 (1.0, 1.02)	<0.001	1.01 (0.99, 1.02)	0.169
Lactates (mmol/L)	0.95 (0.92, 0.97)	<0.001	0.96 (0.93, 0.99)	0.004

BMI, body mass index; PCI, percutaneous coronary intervention; MI, myocardial infarction; IABP, intra-aortic balloon pump; PH, potential of hydrogen; MAP, mean arterial pressure.

### 3.2 Primary and Secondary End Points

**Fig. 2.**
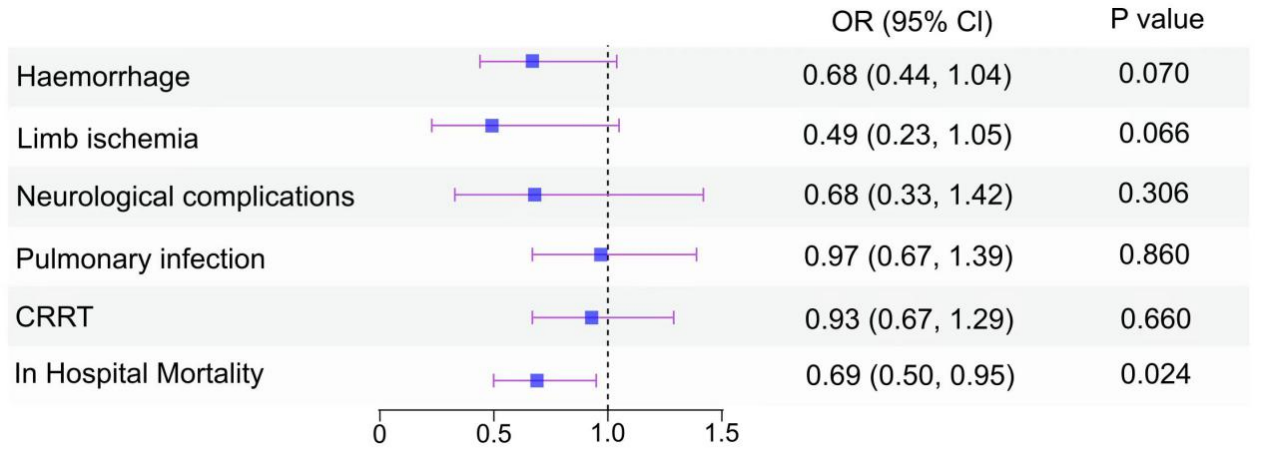
**Forest plot of the OR (95% CI) from multivariable logistic modeling examining the association of PCI and outcomes in AMICS patients assisted by ECMO (after matching)**. Haemorrhage: Data were adjusted for age, BMI, gender, previous PCI, hyperlipidemia, IABP, lactates. Limb ischemia: Data were adjusted for age, BMI, gender, previous PCI, previous MI, smoker, hyperlipidemia, hypertension, diabetes, cardiac arrest, IABP, lactates, MAP. Neurological complications: Data were adjusted for age, BMI, gender, previous PCI, cardiac arrest, IABP, lactates, MAP. Pulmonary infection: Data were adjusted for age, BMI, gender, previous PCI, smoker, hyperlipidemia, hypertension, cardiac arrest, IABP. CRRT: Data were adjusted for age, BMI, gender, previous PCI, hyperlipidemia, heart failure, cardiac arrest, IABP, lactates, MAP. In-hospital mortality: Data were adjusted for age, BMI, gender, previous PCI, diabetes, cerebrovascular accident, chronic respiratory disease, chronic kidney disease, cardiac arrest, IABP, lactates, MAP. OR, odds ratio; CI, confidence interval; AMICS, AMI-related CS; ECMO, extracorporeal membrane oxygenation; BMI, body mass index; PCI, percutaneous coronary intervention; MI, myocardial infarction; IABP, intra-aortic balloon pump; MAP, mean arterial pressure; CRRT, continuous renal replacement therapy; CS, cardiogenic shock; AMI, acute myocardial infarction.

The overall all-cause in-hospital mortality rate was 47.8% (776/1623). The mortality rates, VA-ECMO duration, hospital stay, weaning of ECMO and complication incidences were compared between the PCI and no-PCI groups (Table 3). In the propensity score-matched analysis, the all-cause in-hospital mortality was significantly reduced for the PCI group compared to the no-PCI group, with mortality rates of 40.3% and 51.6% for the PCI and no-PCI group, respectively (*p* = 0.005). The incidence of limb ischemia was significantly lower among patients in the PCI group compared to those in the no-PCI group (3.4% versus [vs.] 7.5%; *p* = 0.036). Patients in the no-PCI group have a higher risk of bleeding (14.1% vs. 20.9%). The impact of PCI on neurological complications, pulmonary infection and CRRT was not statistically significant in the univariate analysis. To account for confounding factors post-matching, a logistic multivariate regression analysis was conducted. All-cause in-hospital mortality was significantly lower in the PCI group than that in the no-PCI group (adjusted odds ratio [aOR] 0.69, 95% confidence interval (CI) 0.50–0.95; *p* = 0.024). Multivariate analysis revealed that PCI treatment didn’t significantly decrease the incidence of lower limb ischemia, potentially due to the utilization of an IABP. Overall, studies have shown that PCI does not increase the incidence of ECMO-related complications (Fig. 2).

**Table 3.** **Outcomes in patients with acute myocardial infarction and cardiogenic shock supported by extracorporeal membrane oxygenation (chi-square test)**.

Outcomes			Total	PCI	No-PCI	*p*
Unmatched groups						
	VA-ECMO duration (hours)		102.5 (33.5, 219.5)	75.8 (26.3, 144.0)	64.6 (20.1, 130.2)	<0.001
	Hospital-stay (days)		12.0 (4.0, 20.0)	13.0 (7.0, 21.0)	10.0 (3.0, 20.0)	0.005
	Weaning of ECMO		1312 (80.8)	568 (88.6)	744 (75.8)	<0.001
		Haemorrhage, n (%)	272 (16.8)	87 (13.6)	185 (18.8)	0.005
		Limb ischemia, n (%)	73 (4.5)	18 (2.8)	55 (5.6)	0.010
	Neurological complications, n (%)		86 (5.3)	27 (4.2)	59 (6.0)	0.140
	CRRT, n (%)		624 (38.4)	230 (35.9)	394 (40.1)	0.095
	Pulmonary infection, n (%)		335 (20.6)	141 (22.0)	194 (19.8)	0.286
	In-hospital mortality, n (%)		776 (47.8)	230 (35.9)	546 (55.6)	<0.001
Matched groups						
	VA-ECMO duration (hours)		87.4 (38.0, 154.0)	83.7 (34.0, 153.4)	93.8 (41.8, 156.4)	0.455
	Hospital-stay (days)		12.0 (6.0, 21.0)	12.0 (6.3, 21.0)	12.0 (5.3, 22.0)	0.891
	Weaning of ECMO		537 (83.9)	276 (86.3)	261 (81.6)	0.107
		Haemorrhage, n (%)	113 (17.7)	46 (14.4)	67 (20.9)	0.038
		Limb ischemia, n (%)	35 (5.5)	11 (3.4)	24 (7.5)	0.036
	Pulmonary infection, n (%)		173 (27.0)	81 (25.3)	92 (28.8)	0.373
	Neurological complications, n (%)		33 (5.2)	13 (4.1)	20 (6.3)	0.283
	CRRT, n (%)		281 (43.9)	134 (41.9)	147 (45.9)	0.339
	In-hospital mortality, n (%)		294 (45.9)	129 (40.3)	165 (51.6)	0.005

VA-ECMO, venoarterial extracorporeal membrane oxygenation; CRRT, continuous renal replacement therapy; PCI, percutaneous coronary intervention.

### 3.3 Survival Analysis

**Fig. 3.**
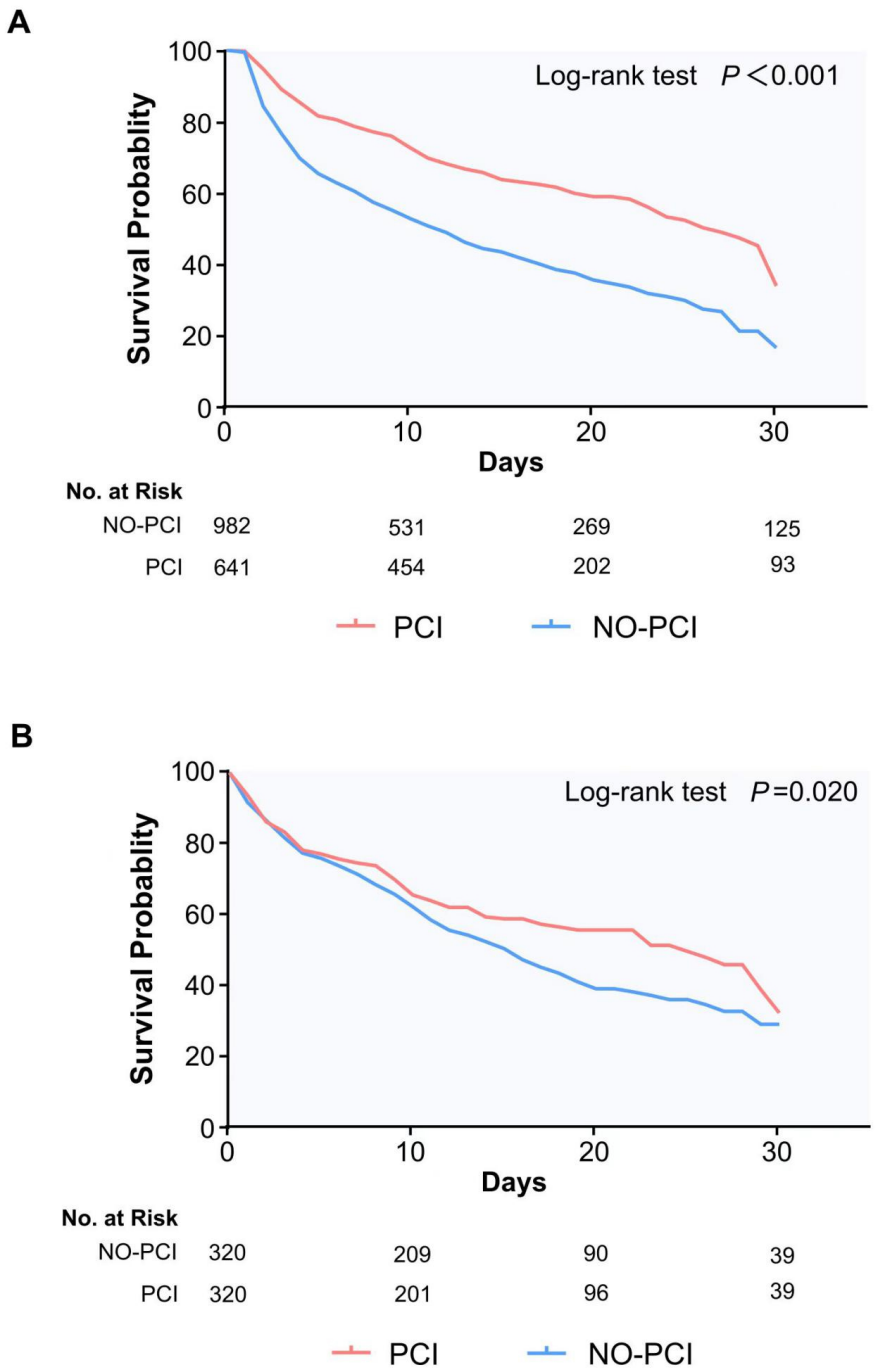
**Kaplan-Meier survival curve, panels (A) and (B) respectively show the 30-day survival of patients before and after propensity score matching**. PCI, percutaneous coronary intervention; No. at Risk, number at risk.

Using Kaplan–Meier analysis, we compared survival rates within 30 days among the PCI and non-PCI cohorts, witnessing a noteworthy disparity in survival chances before and after matching (*p* < 0.001 vs. *p* = 0.020 [log-rank test]) (Fig. 3). The 30-day survival rates for the PCI and non-PCI groups before matching were 57.5% and 38.0%, respectively. After matching, the 30-day survival rates of the two groups were 52.1% and 42.5%.

## 4. Discussion

Cardiovascular (CV) disorders are the leading cause of death worldwide. The most common type of CV disorder is coronary artery disease [3]. Ventricular failure consequent to AMI is the leading cause of CS, comprising over 80% of all cases [16]. Previous studies have reported that, among patients with AMICS, early revascularization of culprit vessels through PCI can improve patient outcomes [5, 17]. Thanks to advancements in revascularization techniques, the mortality rate of patients with AMICS has declined from 70%~80% to 40%~50% [17, 18]. Given the rising incidence of AMICS increases, the mortality reduction achieved through revascularization seems to have reached a plateau [19]. With improvements in living standards and healthcare, the aging population continues to increase. Elderly individuals are more likely to have comorbidities and multivessel CAD. The presence of multivessel CAD is a risk factor for the development of CS [9]. In contrast, the rising incidence of CS related to non-ST-segment elevation myocardial infarction (STEMI) also contributes to the growing number of AMICS cases [20, 21].

Stable patients presenting with risk factors for shock (stage A) or exhibiting early signs of shock (stage B) usually undergo direct coronary angiography followed by revascularization of the culprit vessel. Patients presenting with shock (stages C–E) often necessitate immediate stabilization, focusing on blood pressure management, ensuring adequate end-organ perfusion, oxygenation support, and addressing acid-base imbalances [22, 23]. The compromised general condition, intricate coronary anatomy, and tenuous hemodynamic stability in patients with AMI and CS often complicate the achievement of successful revascularization. Recent advancements in percutaneous mechanical circulatory support (MCS) devices have rendered it feasible to offer hemodynamic assistance to patients suffering from AMI [24, 25, 26, 27, 28]. ECMO can prevent hypotension and reduce cardiac workloads. Notably, even during the advanced stage of cardiogenic shock (Phase E), it offers high-flow comprehensive support, ensuring adequate oxygenation and simultaneous assistance to both the right and left ventricles, thereby granting the necessary time to perform PCI effectively [9, 28]. However, four RCTs and a large meta-analysis of these RCTs does not suggest any survival benefit with the early routine use of VA-ECMO in patients with infarct-related CS compared with optimal medical therapy alone [29, 30, 31, 32, 33]. Our skepticism regarding these findings stems from the largest available trial, the Extra Corporeal Life Support (ECLS)-SHOCK trial, where 26% of patients in the control group received MCS [32]. This potentially could have influenced the outcome and should be considered when interpreting the results. Given that 78.1% of patients in the ECLS group received ECMO either during or following revascularization, it suggests that a substantial proportion of this cohort might have been in a state of shock prior to revascularization without MCS in place. Is it reasonable to initiate MCS when hemodynamics improve following revascularization? Meanwhile, only 5.8% of patients in the ECLS group underwent active left ventricular unloading during ECLS therapy, which is inconsistent with the recognized ECMO management strategy [34]. The benefits of ECMO in such patient populations cannot be overlooked, and a subgroup analysis remains essential to discern the specific groups that derive the most advantage from ECLS.

A total of 1623 eligible patients were included in the present study, of whom 39.5% underwent PCI. The low PCI rate observed in this study is consistent with real-world scenarios [10, 11, 21, 35]. For example, 1 study found that the incidence of PCI in an RCT was higher than that in registered patients (97.5% vs. 58%). This may be because the patients in the RCT were not as ill and more likely to undergo active treatment [10]. This study also found that current PCI treatment tends to be performed in patients with better general conditions (less comorbidities, previous PCI and lower lactate). Although ECMO can provide hemodynamic support for patients with AMICS, a survey addressing the application of ECMO in patients with AMI in the United States found that only 39.3% underwent PCI [11]. A retrospective cohort study conducted in Ontario, Canada, based on a population treated with AMICS, reported that only 44.3% of patients underwent PCI and 21.0% underwent CABG [35]. Furthermore, compared to STEMI, non-STEMI cases have a significantly lower probability of undergoing PCI (84.2% vs. 35.3%; *p* < 0.0001) [21]. Although several pieces of literature recommend interventional treatment for AMICS patients, the PCI implementation rate shown in the above data did not meet expectations [8, 9, 15, 36].

This retrospective study identified a cohort of patients characterized by the presence of severely debilitating diseases. According to the results of univariate analysis before matching, it can be found that patients who have not undergone PCI treatment have a more severe condition. High comorbidity burden, higher incidences of cardiac arrest, and more severe conditions before ECMO support can all influence physicians’ decision-making processes [21]. Upon performing logistic multivariate regression analysis, we observed that the majority of patients who underwent PCI treatment were generally free of hyperlipidemia and chronic respiratory disease, exhibited lower lactate levels, and had a history of prior PCI interventions and smoking. The lower lipids can reduce the burden of atherosclerosis, which may be conducive to improving survival. The lower lactic acid levels indicate a less severe illness. Patients with a history of PCI appear to fare significantly better compared to those without a PCI history. It may be hypothesized that the former group of patients were more likely to be on cardioprotective medications due to their PCI history [19]. In a multivariate analysis, the occurrence of cardiac arrest appears to have minimal influence on PCI treatment decisions. This might be because lactate levels serve as a more indicative marker of the patient’s condition severity. In this study, baseline characteristics were all included as matching indicators to control for potential confounding factors.

After matching, the mortality rate in the PCI group was lower than that in the no-PCI group (40.3% vs. 51.6%, respectively). Observing the data after matching, we note that the comorbidities and pre-ECMO physiological parameters between the two groups are largely balanced. However, there are still some imbalanced factors such as gender, age, previous PCI and IABP. To control for these confounding variables, a logistic multivariate regression analysis was conducted on the matched cohort. We can see that PCI is associated with reducing mortality in patients with AMICS assisted by ECMO (aOR: 0.69; 95% CI 0.50–0.95; *p* = 0.024). PCI is associated with lower mortality rates among critically ill patients. Although this finding is not novel, it highlights the fact that revascularization therapy is crucial for the survival of patients with AMICS supported by ECMO. The CULPRIT-SHOCK trial found that immediate multivessel PCI was associated with a higher risk of death at 30 days than culprit-only PCI with the option of staged revascularization of non-culprit lesions in patients with AMICS [37]. Research has shown that among patients with AMI and refractory CS who received VA-ECMO, residual ischaemia was associated with an increased risk of 1-year mortality [38]. Unfortunately, due to the lack of coronary angiography results, this study was unable to provide recommendations on which blood vessels should undergo PCI treatment. From the univariate analysis, PCI appeared to lessen the risk of limb ischemia. Nevertheless, this protective effect was not confirmed when assessed through multivariate analysis. After the matching process, 61.6% of patients in the non-PCI group relied on IABP, compared to a slightly lower proportion of 40.6% in the PCI group who likewise utilized IABP support. Research [39] has shown that the use of IABP is strongly associated with the occurrence of limb ischaemia. In the cohort of matched patients, the utilization rate of IABP was significantly higher in the non-PCI group compared to the PCI group, which correspondingly increased the risk of limb ischaemia. After incorporating IABP into multivariate analysis, PCI treatment had no significant effect on limb ischaemia. At the same time, the study found that PCI did not increase the incidence of other common complications, such as bleeding, neurological complications, respiratory infection, and acute kidney injury during ECMO assistance.

Current investigations into acute myocardial infarction cases managed with ECMO often center on patients who are scheduled for PCI. However, these studies frequently ignore the situation of real-world critically unwell patients who do not ultimately undergo revascularization procedures. More investigators are concerned about the most appropriate timing for initiating ECMO assistance [27, 36, 40]. Some studies have shown that ECMO before PCI can improve the outcomes of AMICS patients [24, 36]. However, a meta-analysis has examined the influence of the timing of ECMO initiation on PCI, indicating minimal or no significant effect [36]. In this study, the majority of patients received ECMO prior to undergoing PCI, whereas a minority of cases had an indeterminable sequence of PCI and ECMO. A prior study indicated that in cases of AMICS, the emphasis lies on stabilizing hemodynamics, and PCI does not notably enhance survival rates [41]. We maintain that this viewpoint is misguided. Expedited revascularization of the culprit vessel has consistently been the fundamental principle in managing AMICS, be it with or without ECMO support, regardless of the timing of ECMO initiation. PCI of infarct-related arteries is the recommended reperfusion method for patients with AMICS, regardless of the time delay [42]. The aim of the treatment strategy is to promptly initiate ECMO in order to facilitate proper coronary artery and systemic perfusion. This should be accomplished without sacrificing timely revascularization procedures. The goal is to intervene before irreversible organ damage can set in, thereby maximizing the therapeutic advantages of MCS for the patient’s benefit. The significance of this study is that more aggressive revascularization treatment is helpful in reducing the mortality of critically ill patients with AMICS who require ECMO assistance.

### Limitations

This study had some limitations, the first of which was its retrospective observational design and inherent methodological flaws; however, the literature regarding patients treated with ECLS and revascularization is scarce. Although confounding factors were analyzed using multivariate logistic regression analysis and propensity score matching, other unknown confounding factors could not be excluded. Second, due to the lack of coronary angiography results, the description of the characteristics of the population undergoing PCI treatment in this study is too simple. Given the insufficient information on coronary angiography, a thorough evaluation of the treatment outcomes between culprit-only PCI and immediate multivessel PCI is not feasible. Future studies are needed to evaluate the efficacy and safety of immediate multivessel PCI for AMICS supported by ECMO. Third, in a few cases, the sequence of PCI and ECMO procedures is unclear, potentially impacting the accuracy of the analysis. Future research should conduct relevant subgroup analysis. Finally, this study only compared 30-day mortality rates and lacked long-term prognostic data. A study investigating the prognosis of patients with AMICS found that the use of MCS was associated with higher mortality rates, which may be the result of selection bias because patients receiving MCS often have greater disease severity [32]. As such, it pushes for a longer time-dependent analysis (at least 1 year) to determine whether the presumed effect is consistent.

## 5. Conclusions

This is a study exploring whether PCI treatment benefits patients with AMICS supported by ECMO. We found that current PCI treatment tends to be performed in patients with better general conditions (less comorbidities, previous PCI and lower lactate). PCI is associated with lower hospitalization-related mortality in patients with AMICS supported by ECMO. Meanwhile, performing PCI treatment dose not increase the risk of ECMO-related complications. Given ECMO’s substantial role in stabilizing hemodynamics, we recommend that physicians adopt a more aggressive revascularization strategy for patients with AMICS supported by ECMO. Additional research is warranted to assess the effectiveness and risk of performing multi-vessel PCI promptly following ECMO-assisted AMICS. Simultaneously, there is a pressing demand for more comprehensive long-term outcome investigations concentrating on ECMO-assisted AMICS patients who undergo PCI procedures.

## Data Availability

The processed data required to reproduced the above findings cannot be shared at this time due to legal reasons.
